# Supplementary figures and images for: A Compendium of AR Splice Variants in Metastatic Castration-Resistant Prostate Cancer
Source: Int J Mol Sci. 2023 Mar 22;24(6):6009. doi: 10.3390/ijms24066009 (PMC10053078; doi:10.3390/ijms24066009)

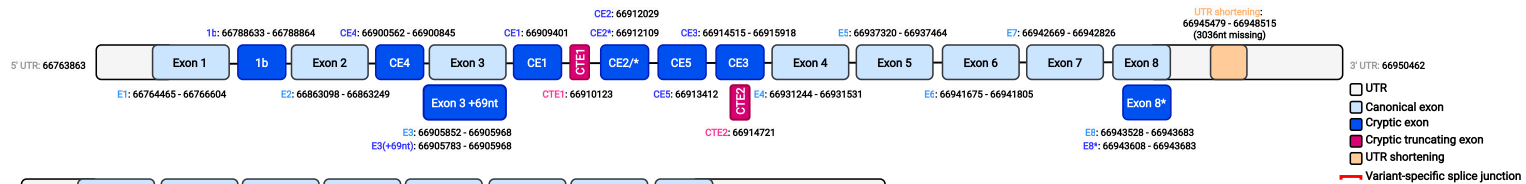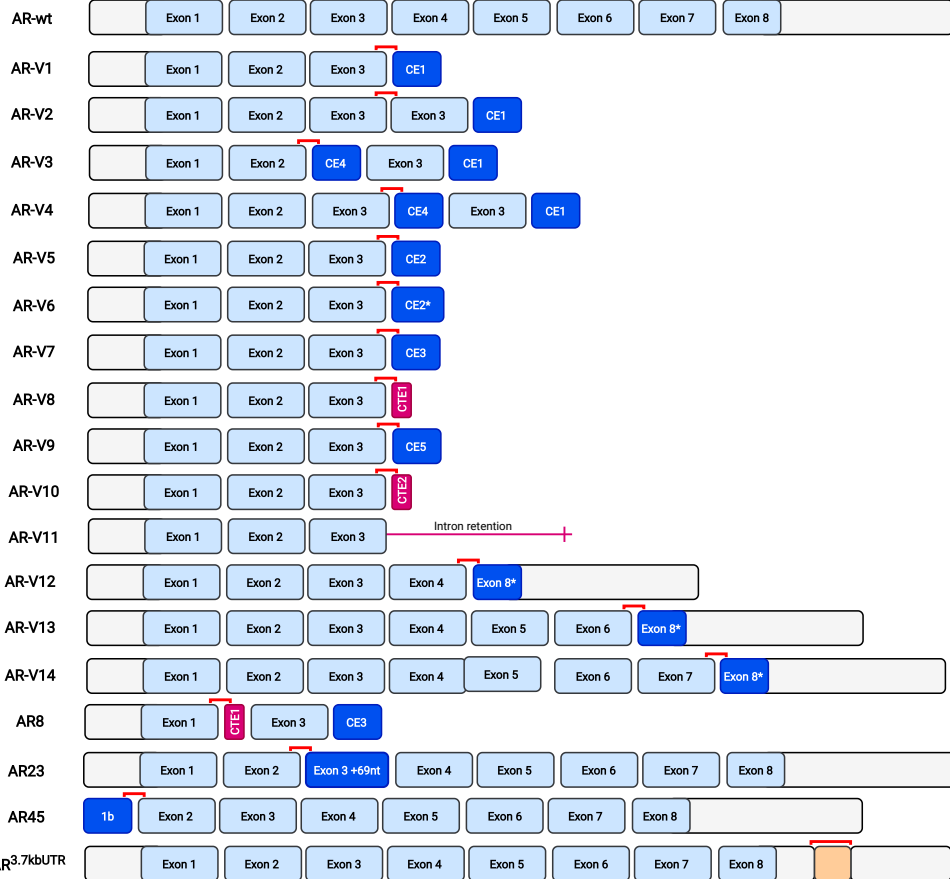

Supplement: Supplementary file 1 [file ijms-24-06009-s001.zip › Figure S1_SplicingLandscapeAR.pdf]
